# Assessment of the impact of intravenous antibiotics treatment on gut microbiota in patients: Clinical data from pre-and post-cardiac surgery

**DOI:** 10.3389/fcimb.2022.1043971

**Published:** 2023-01-19

**Authors:** Ling Xue, Yinglong Ding, Qiong Qin, Linsheng Liu, Xiaoliang Ding, Yi Zhou, Kun Liu, Rajeev K. Singla, Ke Shen, Ahmad Ud Din, Yan Zhang, Zhenya Shen, Bairong Shen, Liyan Miao

**Affiliations:** ^1^ Department of Pharmacy, The First Affiliated Hospital of Soochow University, Suzhou, China; ^2^ Department of Pharmacology, Faculty of Medicine, University of the Basque Country UPV/EHU, Leioa, Spain; ^3^ Department of Cardiovascular Surgery, the First Affiliated Hospital of Soochow University, Suzhou, China; ^4^ Institutes for Systems Genetics, Frontiers Science Center for Disease-related Molecular Network, West China Hospital, Sichuan University, Chengdu, China; ^5^ Reproductive Medicine Centre, The First Hospital of Lanzhou University, Lanzhou, China; ^6^ School of Pharmaceutical Sciences, Lovely Professional University, Phagwara, Punjab, India; ^7^ National Clinical Research Center for Hematologic Diseases, The First Affiliated Hospital of Soochow University, Suzhou, China; ^8^ Institute for Interdisciplinary Drug Research and Translational Sciences, Soochow University, Suzhou, China

**Keywords:** antibiotics, gut microbiota, infection, cardiac surgery, host-microbiome interaction, microbiome

## Abstract

**Background and aims:**

Surgical site infection is a common complication after surgery. Periprocedural antibiotics are necessary to prescribe for preventing or treating infections. The present study aimed to explore the effect of intravenous antibiotics on gut microbiota and menaquinone biosynthesis in patients, especially in elderly patients undergoing cardiac surgery.

**Methods:**

A total of 388 fecal samples were collected from 154 cardiac surgery patients. The V3–V4 hypervariable region of the bacterial 16S rRNA gene was amplified and sequenced on a MiSeq PE300. The gut microbiota diversity of samples was analyzed in terms of α- and β-diversity at the OTU level. The different groups were classified according to antibiotics in combinations and single antibiotics. PICRUSt2 was used for preliminary prediction of the gut microbiota function for menaquinone biosynthesis.

**Results:**

The intravenously administered antibiotics which are excreted via bile represents the main antibiotics that could disturb the gut microbiota’s composition in cardiac surgery patients, especially for elderly patients. The effect of antibiotics on gut microbiota is produced after antibiotics treatments over one week. The recovery of gut microbiota to the state of pre-antibiotics may require over two weeks of antibiotics withdrawal. Sex factor doesn’t represent as an influencer in gut microbiota composition. Long-term use of cefoperazone-sulbactam may affect coagulation function.

**Conclusions:**

The composition of the gut microbiota had a significant change post-intravenous antibiotics treatment in cardiac surgery patients. The richness and diversity of gut microbiota are increased in elderly patients.

## Introduction

Infections are the most common noncardiac complications observed after cardiac surgery (Gelijns et al., 2014; Liu et al., 2021). Surgical site infection (SSI) of the sternum is a common complication following cardiac surgery (Gudbjartsson et al., 2016). The incidence of SSI in cardiac surgery is across a broad range. This complication is associated with an increase in the duration of hospitalization, mortality, and economic burden (Frenette et al., 2016; Gudbjartsson et al., 2016; Schimmer et al., 2017; Kachel et al., 2020; Liu et al., 2021). The incidence of readmission and mortality for SSI patients is about 46% and 1-14%, respectively, and the cost of treatment is 2.5 times higher than for non-SSI patients (Frenette et al., 2016; Gudbjartsson et al., 2016). It was reported the incidence of mortality was about 23.6% for Chinese patients with nosocomial infections, which was higher than patients without nosocomial infections (2.3%) (Liu et al., 2021). The prevention of post-cardiac surgery infection remains a major challenge. Clinical practice guidelines for antibiotic prophylaxis provide some recommendations in cardiac surgery (Hamouda et al., 2015; Eckardt et al., 2018). Periprocedural antibiotics are necessary to prevent infection post-cardiac surgery (Gudbjartsson et al., 2016; Schimmer et al., 2017; Kachel et al., 2020; Surat et al., 2021).

The surface and inside of humans are covered by a multitude of microorganisms. The gastrointestinal tract is the site of the majority of microbial biomass and biodiversity in the human body (Bibbo et al., 2016; Jourova et al., 2016; Gough, 2022). Recently, gut microbiota was found to play a critical role in many physiological and pathological processes in the human body. The physiology of almost all organisms’ organs is influenced by gut microbiota. In particular, gut microbiota could influence the biotransformation of xenobiotics (Lozupone et al., 2012; Jourova et al., 2016; Gough, 2022). However, the use of antibiotics could disturb the composition and functioning of the gut microbiota (Becattini et al., 2016; Jourova et al., 2016; Mangiola et al., 2018; Hao et al., 2020; Gough, 2022). Some studies evaluated the impact of antibiotics on the gut microbiota in pediatric patients. Oral antibiotics were the major intervention for part of these studies (Gough, 2022). Although some intravenous antibiotics also were evaluated, a single antibiotic was the intervention in most studies (Gough, 2022). However, the clinical use of antibiotics is either in the form of combination therapy or frequent replacement of antibiotics. In addition, antibiotic drugs can affect intestinal bacteria only when antibiotic drugs are circulated to the gut in theory. Therefore, oral antibiotics could theoretically affect the composition of gut bacteria, while only intravenous antibiotics which could excrete *via* the biliary tract and reaches the intestine to influence gut bacteria. Few studies have evaluated the effect of combined antibiotics on gut bacteria in clinical practice. It has been demonstrated that antibiotic use is associated with neurological disorders that range from anxiety and panic attacks to major depression, psychosis, and delirium in humans (Angelucci et al., 2019). This may be related to interference with the gut microbiota by antibiotics (Angelucci et al., 2019). Changes in the composition of the gut microbiota should also be considered in patients with psychiatric disorders after surgery.

The purposes of the present study were to explore the onset time of intravenous antibiotics on gut microbiota and the time of recovery of gut microbiota to the state of pre-antibiotic after antibiotic withdrawal and to explore the effect of gut microbiota on menaquinone biosynthesis in patients, especially in elderly patients undergoing cardiac surgery.

## Materials and methods

### Patients and sampling

The present study was approved by the health authority ethics committee of the First Affiliated Hospital of Soochow University and as per the Declaration of Helsinki. All patients gave written informed consent. Patients underwent cardiac surgery for a variety of reasons, including valvular heart disease, rheumatic valve disease, congenital heart disease, etc.

These patients were administered antibiotics by intravenous drip for the prevention and/or treatment of infection. Generally, cefuroxime was administered first by intravenous drip before 0.5-1 h of surgery. Then, a replacement or combination with other antibiotics was performed by surgeons according to the patient’s condition. The used antibiotics included cefuroxime, ceftazidime, cefoperazone-sulbactam (sulperazone), imipenem cilastatin (tienam), meropenem, levofloxacin, moxifloxacin, ciprofloxacin, vancomycin, teicoplanin, tigecycline, ticarcillin-clavulanate, piperacillin-tazobactam, and penicillin. The regimen of combined antibiotics included: sulperazone combined with quinolones (levofloxacin, moxifloxacin, ciprofloxacin) or glycopeptides (teicoplanin, vancomycin); carbapenems (tienam, meropenem) combined with quinolones, glycopeptides or tigecycline, etc.

Fecal samples of patients were collected to analyze the composition of the gut microbiota. The time of fecal sample collection was set as: before antibiotics administration (pre-antibiotic group), antibiotics administration over 7 days (post-antibiotic group), and antibiotics withdrawal over 7 days (withdrawal-antibiotic group) for cardiac patients.

In order to evaluate the stability of fecal samples at room temperature, fecal samples by three healthy volunteers were kept at room temperature for 0, 2, 4, and 6 h after defecation.

All fecal samples were collected into a sterile tube (Sarstedt, Germany) and then stored at -80°C until further analysis.

### Gut microbiota analysis

Bacterial genomic DNA was extracted from fecal samples using a genomic DNA purification kit (Omega Biotek, USA) following the manufacturer’s protocol. The V3–V4 hypervariable region of the bacterial 16S rRNA gene was amplified by polymerase chain reaction (PCR) using the primer pairs: 338F, 5’-ACTCCTACGGGAGGCAGCAG-3’, and 806R, 5’-GGACTACHVGGGTWTCTAAT-3’. PCR reaction system included: 4μL 5×fastpfu buffer, 2μL 2.5 mM deoxyribonucleotide triphosphates (dNTPs), 0.8μL 5μM primer, 0.4μL fastpfu polymerase, 0.2μL bovine serum albumin (BSA) and 10 ng template DNA. The remaining volume would be supplemented with purified water up to 20µL. The PCR cycling conditions were as follows: 3 min at 95°C; 27 cycles of the 30s at 95°C, 30s at 55°C, and 45s at 72°C; and a final extension step at 72°C for 10min. PCR was conducted in triplicate for each sample using an ABI GeneAmp^®^ 9700 thermocycler (ABI, USA).

The PCR product was extracted from a 2% agarose gel and purified using the AxyPrep DNA gel extraction kit (Axygen Biosciences, USA) according to the manufacturer’s protocol and quantified using a Quantus™ fluorometer (Promega, USA). Purified PCR product amplicons were pooled in equimolar amounts and paired-end sequenced on MiSeq PE300 (Illumina, USA) according to the standard protocols developed by Majorbio Bio-Pharm Technology Co. Ltd. (Shanghai, China).

The raw 16S rRNA gene sequencing reads were demultiplexed, quality-filtered by FASTP (version 0.19.6) (Chen et al., 2018), and merged by Flash (version 1.2.11) (Magoc and Salzberg, 2011). Operational taxonomic units (OTUs) with a 97% similarity cutoff (Stackebrandt and Goebel, 1994; Edgar, 2013) were clustered using UPARSE (version 7.0.1090) (Edgar, 2013), and chimeric sequences were identified and removed. The taxonomy of each OTU representative sequence was analyzed by the RDP classifier (version 2.11) (Wang et al., 2007) against the 16S rRNA database (Silva v138) using a confidence threshold of 0.7.

### Data analysis in all patients’ population

Firstly, the data was divided into different groups before analysis. The different groups included the classification according to antibiotics (antibiotic treatment), single antibiotic (such as cefuroxime), and replacement antibiotic (such as ceftazidime, sulperazone, tienam, meropenem, and quinolone replaced cefuroxime). The latter two groups were uniformly classified as a single antibiotic group because patients were also prescribed a single antibiotic for the replacement antibiotic group when fecal samples were collected. The difference in gut microbiota was also analyzed between the elderly and the non-elderly (younger) and between the sexes. Elderly was defined as age ≥60 years, and non-elderly was defined as age< 60 years in the present study.

The gut microbiota diversity of samples was analyzed in terms of *α-* and *β-*diversity at the OTU level. The indexes of *Sobs* and *Chao* were used to reflect community richness for *α-*diversity. The indexes of *Shannon* and *Simpson* were used to reflect community diversity for *α-*diversity. The index of *Coverage* was used to reflect community coverage. PCoA (principal coordinates analysis) was used for *β-*diversity analysis, and *Bray-Curtis* and *Anosim* were chosen for the statistical test. The distribution proportion of dominant bacteria in each group of samples and the distribution proportion of each dominant bacteria in different groups were reflected at the genus level by a *Circos* graph.

*Mann–Whitney U* test was used as a statistical test method for *α-*diversity indexes for the antibiotics group by GraphPad (version 8.0). *Wilcoxon signed-rank* test was used as a statistical test method for *α-*diversity indexes for single antibiotic and replacement antibiotic by GraphPad (version 8.0).

The differences in gut microbiota for the top 20 bacterial species which was the relative abundance of bacterial species ranked in the first 20 were compared between the pre-antibiotic and post-antibiotic groups, and between the pre-antibiotic and withdrawal-antibiotic groups by *Mann–Whitney U* test at the genus level in the antibiotics group. The differences in gut microbiota for the top 20 bacterial species were compared between the pre-antibiotic and post-antibiotic groups by *Wilcoxon signed-rank* test in a single antibiotic group. Then, the present study further analyzed the effect of the days of antibiotics administration and withdrawal on gut microbiota at the genus level by the *Wilcoxon signed-rank* test, and this analysis also focused on the top 20 bacterial species in the antibiotics group.

PICRUSt2 (phylogenetic investigation of communities by reconstruction of unobserved states) was used to preliminarily predict the functioning of gut microbiota based on the KEGG (Kyoto encyclopedia of genes and genomes) database. Menaquinone biosynthesis by gut microbiota (www.genome.jp/pathway/map00130+K05357) was the concern in the present study.

### Data analysis in elderly patients’ population

In addition, the difference in gut microbiota for pre- and post-antibiotic treatment also was explored for elderly patients. Only the data for elderly patients who had simultaneously provided the fecal samples in both pre-antibiotic and post-antibiotic stages was analyzed in the current study. The analysis method of gut microbiota diversity in elderly patients was performed in terms of α- and β-diversity at the OTU level. All the selected indexes were same with above for *α*- and *β*-diversity. The differences in gut microbiota for the top 20 bacterial species were compared by *Wilcoxon signed-rank* test in elderly patients.

All data analyzing were performed on the free online platform of the Majorbio Cloud Platform (www.majorbio.com) for the diversity and functioning of gut microbiota.

## Results

### Patients’ characteristics

A total of 154 cardiac surgery patients participated in the present study. The characteristics of the patients are shown in **Table 1**. Most patients underwent heart valve replacement surgery. All patients were administered any specific kind of antibiotic for the prevention or treatment of infection. A total of 388 fecal samples were collected from these patients. The number of fecal samples for the pre-antibiotic, post-antibiotic, and withdrawal-antibiotic groups were 154, 152, and 82, respectively. A summary of antibiotic regimens for the prevention or treatment of infection is shown in **Table 2**.

**Table 1:** Demographic characteristics of the patients.

Items	Value (Mean ± SD or Proportion)
Age (years)	59.8 ± 10.7
Height (cm)	163.1 ± 8.7
Weight (kg)	63.4 ± 9.8
BMI (kg/m^2^)	23.8 ± 2.9
Male	86 (55.8%)
Female	68 (44.2%)
Days 1 (days)	8.4 ± 6.8
Days 2 (days)	11.1 ± 6.0
Days 3 (days)	13.2 ± 9.0
Days 4 (days)	17.9 ± 10.3
Indication of surgery	
Valvular heart diseases (n, %)	99 (64.3%)
Rheumatic valve disease (n, %)	44 (28.6%)
Ascending aortic aneurysm (n, %)	4 (2.6%)
Congenital heart disease (n, %)	4 (2.6%)
Aortic root aneurysm (n, %)	2 (1.3%)
Aortic dissection (n, %)	1 (0.65%)

BMI, Body mass index; Days 1, Preoperative days when fecal samples collection; Days 2, Days of antibiotic use when fecal samples collection; Days 3, Days of antibiotic withdrawal when fecal samples collection; Days 4, Days of antibiotic use during the follow-up period.

**Table 2:** Antibiotic regimen characteristics of antibiotic administration during the follow-up period.

Antimicrobial regimen	Value (n, %)
Cefuroxime	21(13.6%)
Cefuroxime and Ceftazidime	10(6.5%)
Cefuroxime and Sulperazone	49(31.8%)
Cefuroxime and Tienam	13(8.4%)
Cefuroxime and Meropenem	6(3.9%)
Cefuroxime and Quinolones	6(3.9%)
Cefuroxime, Sulperazone, and Tienam	1(0.6%)
Cefuroxime, Sulperazone, and Meropenem	2(1.3%)
Cefuroxime, Sulperazone, and Quinolones	10(6.5%)
Cefuroxime, Sulperazone, Tienam, and Quinolones	2(1.3%)
Cefuroxime, Sulperazone, Vancomycin, and Quinolones	2(1.3%)
Cefuroxime, Sulperazone, and Vancomycin	1(0.6%)
Cefuroxime, Sulperazone, Vancomycin, and Meropenem	1(0.6%)
Cefuroxime, Tienam, and Vancomycin	3(1.9%)
Cefuroxime, Tienam, and Quinolones	2(1.3%)
Cefuroxime, Quinolones, and Meropenem	1(0.6%)
Other	24(15.6%)

Other: Clindamycin, Teicoplanin, Ticarcillin-clavulanate, Tigecycline, Piperacillin tazobactam, and Penicillin.

### Gut microbiota analysis in the antibiotics group for all patients’ population

**Figure 1:**
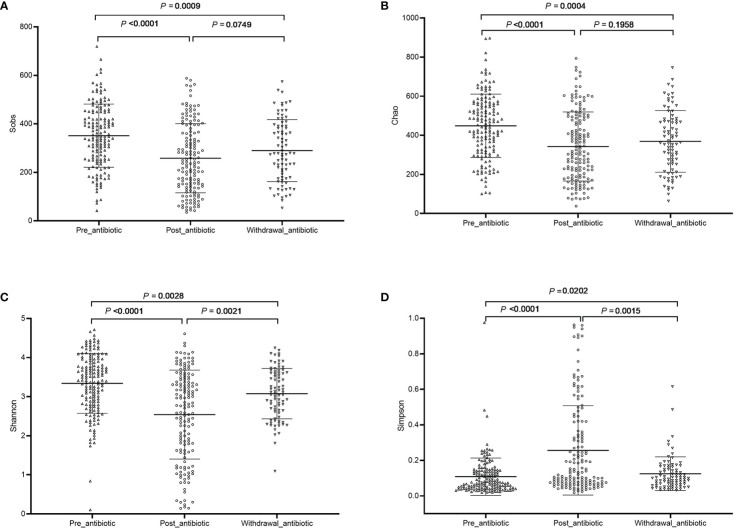
The community richness and diversity of each group of gut microbiota in the antibiotics group. **(A)**: *Sobs* index; **(B)**: *Chao* index; **(C)**: *Shannon* index; **(D)**: *Simpson* index.

The community richness and diversity are shown in **Figure 1**. Compared with the pre-antibiotic group, all the indexes of *α-*diversity were significantly different in the post-antibiotic and withdrawal-antibiotic groups (*P*<0.05). *Sobs* and *Chao* indexes indicated no significant difference between the post-antibiotic and withdrawal-antibiotic groups. However, *Shannon* and *Simpson’s* indexes revealed a significant difference between the post-antibiotic and withdrawal-antibiotic groups. These results indicate that the richness and diversity of gut microbiota are decreased post antibiotics administration which is quite predictable. When antibiotics are withdrawn, the same level of richness may not recover, but diversity has some recovery. However, the diversity could not recover to the same or similar level of pre-antibiotic. All coverage indexes of gut microbiota community coverage were over 0.99.

**Figure 2:**
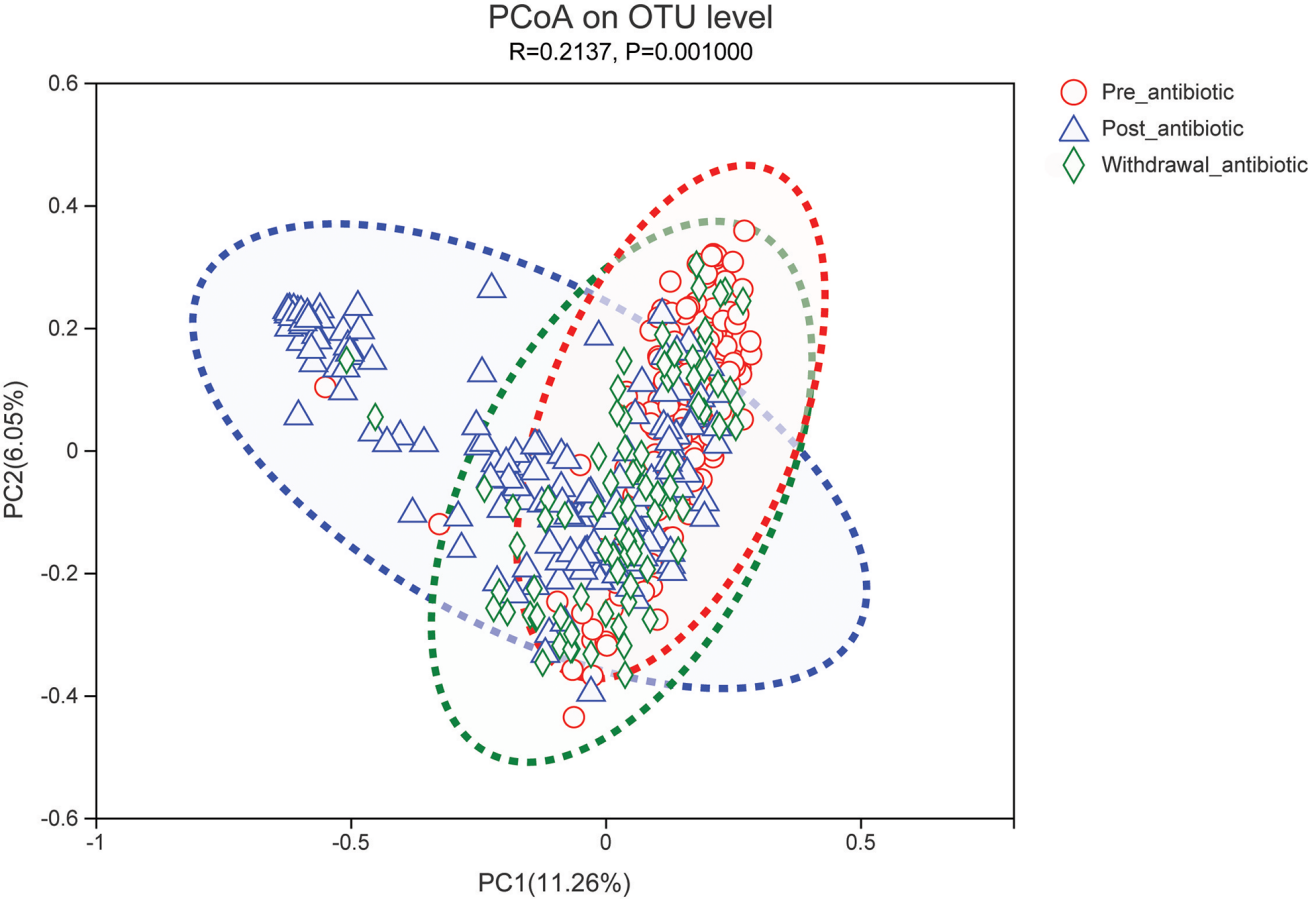
PCoA (principal coordinates analysis) of gut microbiota in each group for the antibiotics group. The dotted line represents the 95% confidence interval line.

**Figure 3:**
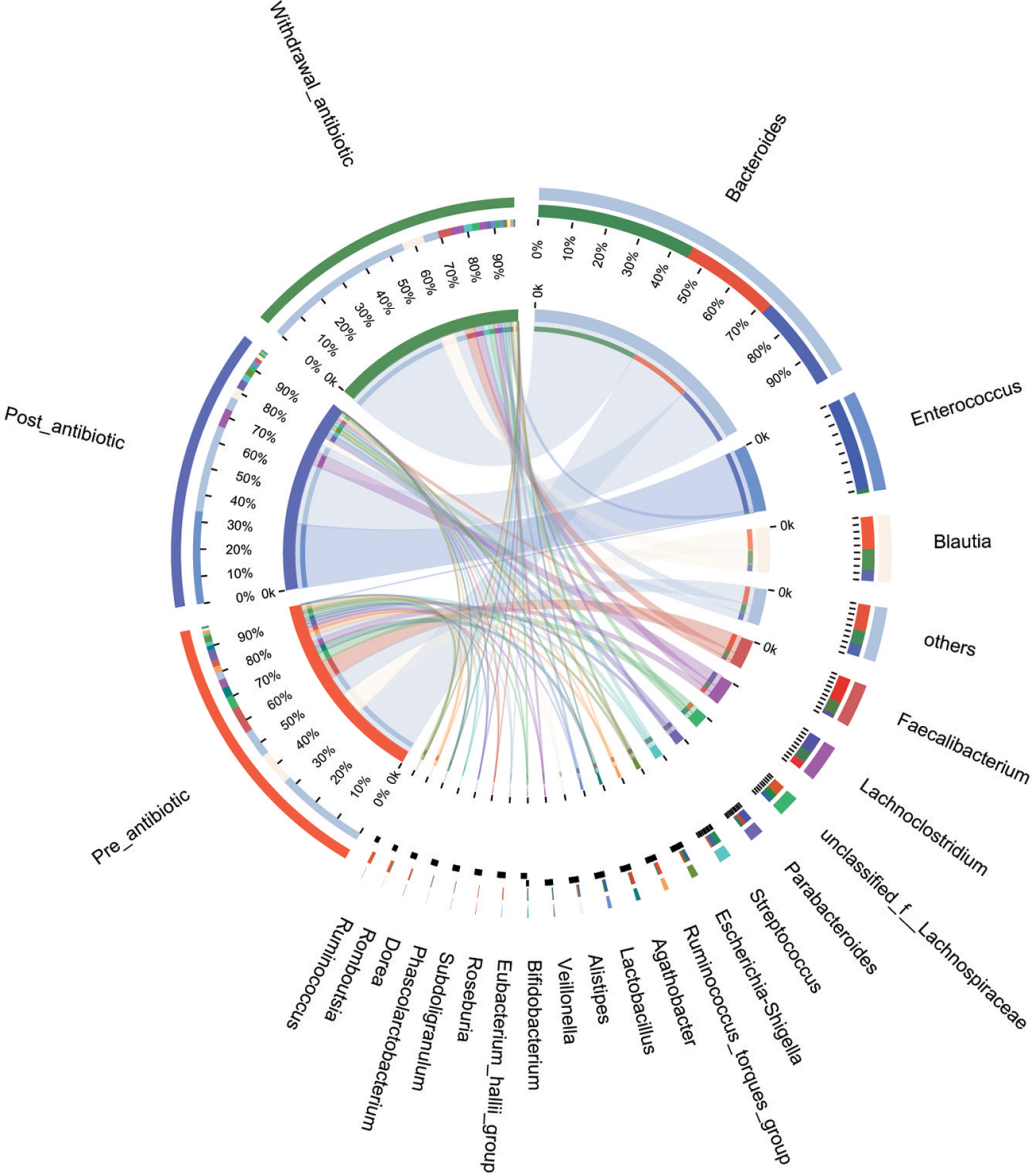
The distribution of dominant bacteria of the sample in each group for the antibiotics group.

PCoA showed that post-antibiotic group had a significant difference from pre-antibiotic and withdrawal-antibiotic groups at the OTU levels in the antibiotics group (**Figure 2**). It also indicates that post-antibiotic over 7 days could significantly affect the *β-*diversity of gut microbiota, and *β-*diversity could recover when antibiotics are withdrawn over 7 days. The distribution of dominant bacteria in each group sample is shown in **Figure 3**. The dominant bacteria included that from *Bacteroides*, *Enterococcus*, *Blautia*, *Faecalibacterium*, *Lachnoclostridium*, etc.

**Figure 4:**
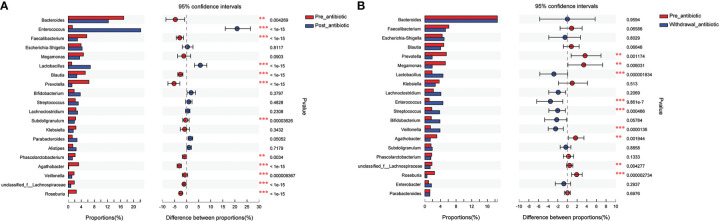
Comparison of the top 20 bacterial species in the antibiotics group. **(A)**: comparison between pre-antibiotic and post-antibiotic; **(B)**: comparison between pre-antibiotic and withdrawal-antibiotic. **P<0.01, ***P<0.001.

Results from fecal samples revealed that 12 bacterial genera had a significant variation when antibiotics were administrated over 7 days, and the relative abundance of *Enterococcus* and *Lactobacillus* increased (*P*<0.05, **Figure 4A**). Even with antibiotics withdrawn over 7 days, 9 bacterial genera had also indicated significant difference (*P*<0.05, **Figure 4B**), however, the relative abundance of *Bacteroides, Faecalibacterium, Blautia*, *Subdoligranulum*, and *Phascolarctobacterium* had no significant variation when antibiotics were withdrawn over 7 days (*P*>0.05, **Figure 4B**).

**Figure 5:**
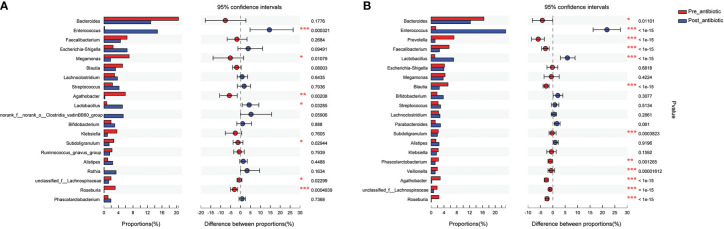
Comparison of the top 20 bacterial species of the effect of antibiotics treatment days on gut microbiota in the antibiotics group. **(A)**: antibiotics treatment for 7 days; **(B)**: antibiotics treatment over 7 days. *P<0.05, **P<0.01, ***P<0.001.

The present study further analyzed the effect of the number of days of antibiotics administration and withdrawal on gut microbiota at the genus level. The antibiotics administration group was divided into 7 days (the number of patients was 19, corresponding fecal samples size was 38) and over 7 days (the number of patients was 133, corresponding fecal samples size was 266) according to the days of antibiotics use. Compared with the pre-antibiotic group, the relative abundance of 7 bacterial genera had significantly changed when antibiotics were administrated for 7 days (*P*<0.05, **Figure 5A**), and the number of the changed bacterial genera significantly increased when antibiotics were administrated more than 7 days (*P*<0.05, **Figure 5B**). This result also showed an increase in the relative abundance of *Enterococcus* and *Lactobacillus*, while the relative abundance of other bacterial genera was decreased in this analysis when antibiotics were administrated for more than or equal to 7 days (*P*<0.05, **Figure 5**).

**Figure 6:**
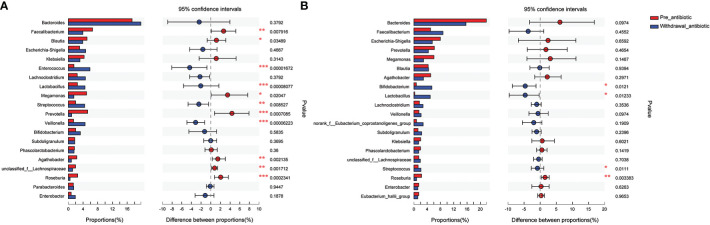
Comparison of the top 20 bacterial species of the effect of antibiotics withdrawal days on gut microbiota in the antibiotics group. **(A)**: antibiotics withdrawal 7-14 days; **(B)**: antibiotics withdrawal over 14 days. *P<0.05, **P<0.01, ***P<0.001.

The antibiotics withdrawal group was divided into less than 14 days (the number of patients was 59, corresponding fecal samples size was 118) and more than 14 days (the number of patients was 23, corresponding fecal samples size was 46) according to the days of withdrawal. Compared with the pre-antibiotic group, the relative abundance of 11 bacterial genera had also been significantly different when antibiotics were withdrawn for 7-14 days (*P*<0.05, **Figure 6A**), and the number of the changed bacterial genera significantly decreased when antibiotics were withdrawn more than 14 days (*P*<0.05, **Figure 6B**). Based on the results, we could assume that the relative abundance of gut microbiota could gradually return to the state of pre-antibiotic except for *Lactobacillus, Streptococcus*, and *Roseburia* when antibiotics were withdrawn for more than 14 days.

### Gut microbiota analysis in the single antibiotic group for all patients’ population

Further, the effects of a single antibiotic on gut microbiota were explored in this study and the samples were from patients who had simultaneously been provided with a single antibiotic in both pre-antibiotic and post-antibiotic stages. The use of antibiotics was generally adjusted according to the patient’s condition as per the clinical practice. The fecal sample data of patients who were prescribed with only a single antibiotic was collected and analyzed. There were patients whose antibiotic regimen for cefuroxime was replaced by ceftazidime, sulperazone, tienam, meropenem, or quinolones in the present study. In order to rationalize the sample size, levofloxacin, moxifloxacin, and ciprofloxacin were analyzed as one class i.e., quinolones. The number of samples for cefuroxime, ceftazidime, sulperazone, tienam, meropenem, and quinolones were 42, 20, 98, 26, 12, and 12, respectively.

The community richness and diversity are shown in **Figures S1**–**S6**. The results revealed that cefuroxime could change the richness (*P*<0.05) and had no effect on diversity (*P*>0.05, **Figure S1**), and sulperazone could change both the richness and diversity (*P*<0.05, **Figure S3**), however, ceftazidime, tienam, meropenem, and quinolones had no effect on *α-*diversity of gut microbiota (*P*>0.05).

PCoA showed that sulperazone, tienam, and meropenem could significantly affect the *β-*diversity (**Figures S7C–E**), while cefuroxime, ceftazidime, and quinolones had no effect on the *β-*diversity of gut microbiota at the OTU levels when these antibiotics were prescribed over 7days (**Figures S7A, B, F**).

The number of varied relative abundance of bacterial genera was 4, 6, 14, 9, 3, and 1 for cefuroxime, ceftazidime, sulperazone, tienam, meropenem, and quinolones, respectively (**Figure S8**). The relative abundance of gut microbiota was increased for *Escherichia-Shigella* and *Akkermansia* by cefuroxime, *Blautia* by ceftazidime and quinolones, *Lactobacillus* by ceftazidime and sulperazone, *Enterococcus* by ceftazidime, sulperazone, tienam and meropenem, and *Bifidobacterium* by tienam.

### Gut microbiota analysis for age and sex for all patients’ population

**Figure 7:**
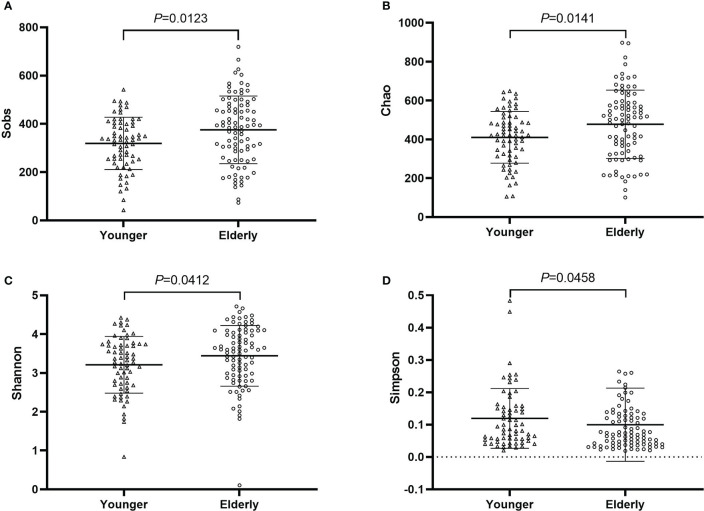
The community richness and diversity of gut microbiota for younger and elderly. **(A)**: *Sobs* index; **(B)**: *Chao* index; **(C)**: *Shannon* index; **(D)**: *Simpson* index.

Fecal samples of pre-antibiotics were used to compare the difference between the elderly and the non-elderly and between the sexes. The number of samples was 88 for the elderly, and 66 for the non-elderly. The richness and diversity of gut microbiota were significantly increased in the elderly (*P*<0.05, **Figure 7**), however, there was no considerable difference between females and males (*P*>0.05, **Figure S9**). PCoA and the top 20 bacterial species also showed no difference between the elderly and the non-elderly and between the sexes (*P*>0.05, **Figures S10**, **S11**).

### Gut microbiota analysis in elderly patients’ population

**Figure 8:**
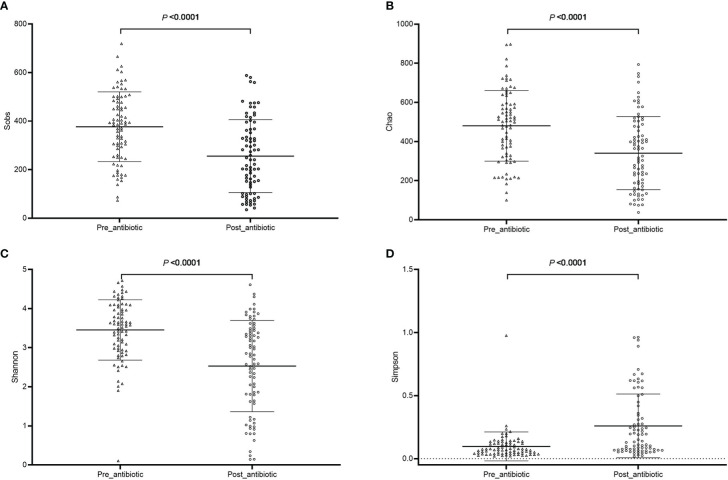
The community richness and diversity of gut microbiota for elderly patients. **(A)**: *Sobs* index; **(B)**: *Chao* index; **(C)**: *Shannon* index; **(D)**: *Simpson* index.

**Figure 9:**
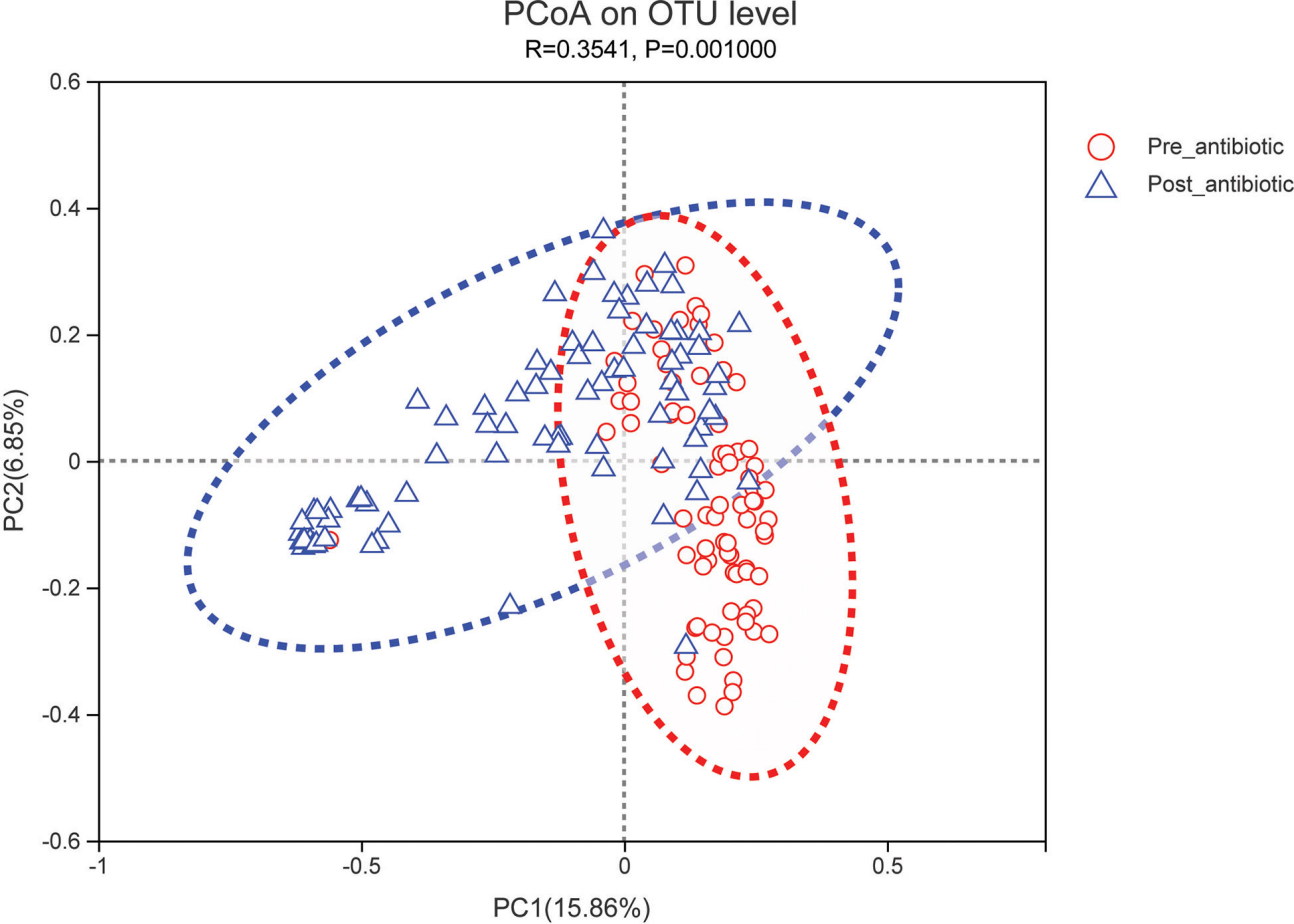
PCoA (principal coordinates analysis) of gut microbiota for elderly patients. The dotted line represents the 95% confidence interval line.

**Figure 10:**
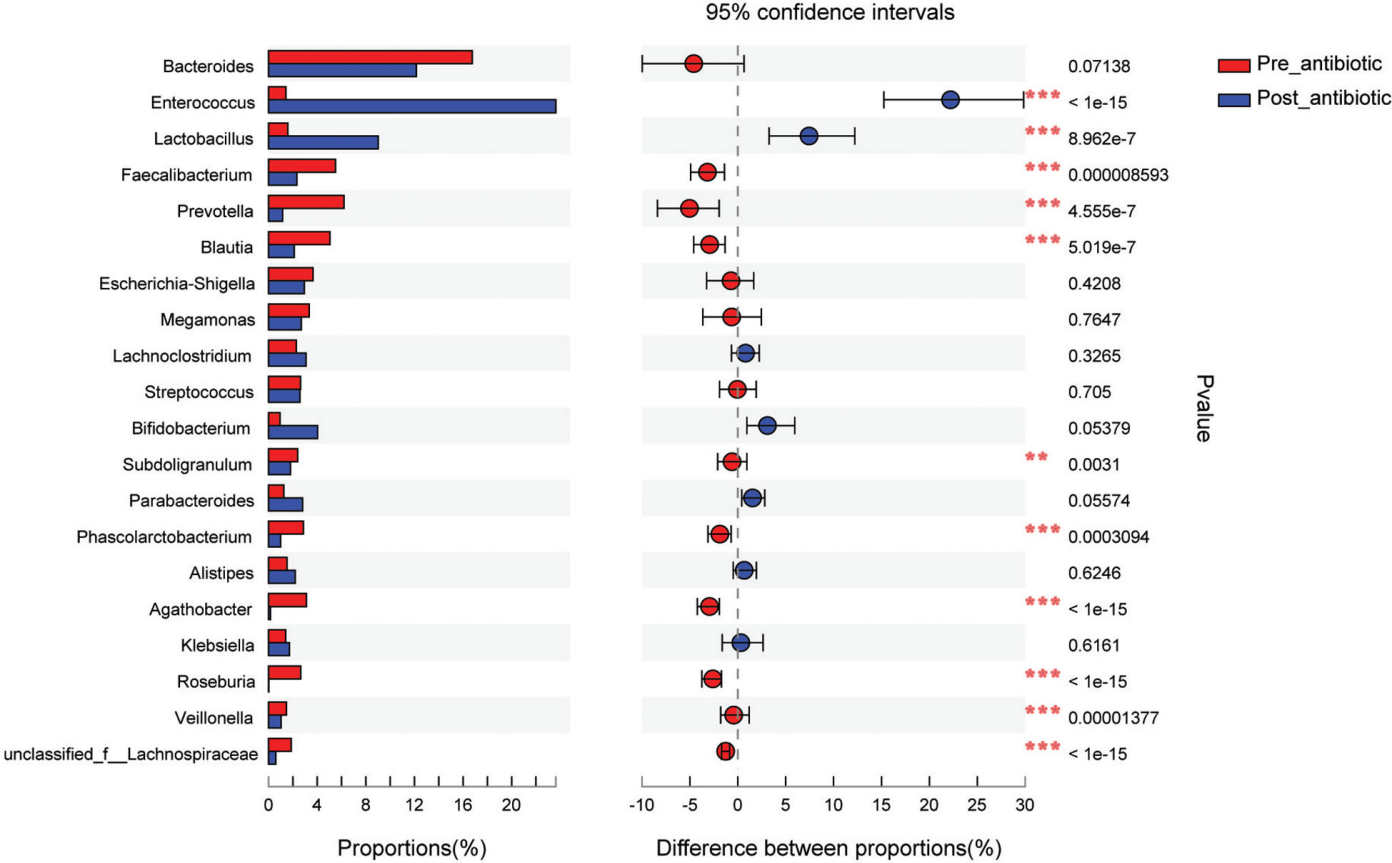
Comparison of the top 20 bacterial species for elderly patients. **P<0.01, ***P<0.001.

The effects of antibiotic treatment on gut microbiota were explored in elderly patients who had simultaneously provided the fecal samples in both pre-antibiotic and post-antibiotic stages. The number of patients was 78, the corresponding fecal samples size was 156. Compared with the pre-antibiotic group, all the indexes of *α*-diversity revealed significantly difference in the post-antibiotic groups (*P*<0.05, **Figure 8**). PCoA showed that post-antibiotic groups revealed a significant difference from pre-antibiotic group at the OTU levels in elderly patients (**Figure 9**). 11 bacterial genera revealed a significant difference post-antibiotic treatment over 7 days, and the relative abundance of *Enterococcus* and *Lactobacillus* increased, while a decrease in the relative abundance of other bacterial genera with a significant difference has been observed (*P*<0.05, **Figure 10**).

### Stability study of fecal samples from healthy volunteers

The stability of the fecal sample was evaluated at room temperature in terms of *α-*, *β-*diversity, and the top 20 bacterial species. The results showed that the *a-* and *β-*diversity at the OTU level and the top 20 for the relative abundance of bacterial species of gut microbiota had no significant difference when the fecal sample was placed for approximately 6 hours at room temperature (*P*>0.05, **Figures S12**–**S14**). It demonstrated that the broader composition of the gut microbiota had no significant change when the fecal sample was stored at room temperature for 6 hours.

### Function prediction analysis of gut microbiota

Menaquinone is mainly synthesized by gut microbiota. The pathway of menaquinone biosynthesis could be found in the KEGG database. The process of menaquinone biosynthesis involves enzymes and intermediate compounds as shown in **Figure S15**. The effect of gut microbiota on the enzymes of menaquinone biosynthesis was explored in a sulperazone- treated group. The result showed that the relative abundance of bacterial genera which involved some enzymes of menaquinone biosynthesis was significantly reduced post-sulperazone treatment over 7 days (**Table 3**).

**Table 3:** Change of the relative abundance of gut bacteria involved enzyme post sulperazone treatment in the process of menaquinone biosynthesis.

Enzyme	Description	Pre-sulperazone (Median, 2.5-97.5% percentiles)	Post-sulperazone (Median, 2.5-97.5% percentiles)	*P* values
2.1.1.163	Demethylmenaquinone methyltransferase	11413.4(1580.3-24135.4)	4351.3 (115.4-28675.1)	0.0100
4.2.1.151	Chorismate dehydratase	654.0 (0.008-16195.8)	32.0 (0.008-25668.4)	0.0132
2.5.1.120	Aminodeoxyfutalosine synthase	818.0 (0.008-31876.4)	36.0 (0.008-51322.2)	0.0109
3.2.2.26	Futalosine hydrolase	68.0 (0.008-1246.2)	5.0 (0.008-518.6)	0.0002
1.21.98.1	Cyclic dehypoxanthinyl futalosine synthase	654.0 (0.008-16195.8)	32.0 (0.008-25668.4)	0.0132
1.14.-.-	1,4-dihydroxy-6-naphthoate synthase	136.0 (0.008-3154.1)	14.0 (0.008-2812.8)	0.0032

## Discussion

Most surgeries require the use of antibacterial drugs to prevent and/or treat infections at the surgical site, including superficial and deep incision infections as well as the organs or cavities involved in the surgery. The non-specific bactericidal effect of antibiotics is mainly influencing the bacterial growth curve of pathogens (Ferrer et al., 2017). Therefore, antibiotic treatment could potentially modulate the composition of gut microbiota (Lozupone et al., 2012; Mangiola et al., 2018; Hao et al., 2020). It is well-recognized that gut bacteria play a crucial role in host homeostasis and are involved directly or indirectly in the progression and development of some diseases and disorders (Mamieva et al., 2022). Generally, the composition of the gut microbiota is relatively stable at the species level during adult life. Although antibiotic treatment might perturb this balance, these perturbations were temporary.

To evaluate the stability of the fecal sample which stayed at room temperature without affecting the composition of gut microbiota, 12 fecal samples were collected from 3 healthy subjects. This work to assess stability study was performed because the fecal samples could not store in a -80°C refrigerator immediately after patients’ defecation in clinical practice. The fecal samples were put into a -80°C refrigerator after defecation within 5 minutes (as 0 h), and kept defecation samples for 2, 4, and 6 hours, respectively at room temperature. All fecal samples except 0-hour related samples were stored at room temperature before putting into a -80°C refrigerator. The result demonstrated that the broader composition of the gut microbiota had no significant change when the fecal sample was stored at room temperature even for 6 hours. It was previously reported that fecal samples should be transferred to the laboratory within 24 h after defecation when stored at room temperature or within 3 days when stored in a refrigerated condition (Al et al., 2018; Nagata et al., 2019). These results demonstrated that the fecal sample after defecation of patients could place at room temperature for a period of time, which has little effect on the change of gut microbiota composition, which meets clinical practice. In the present study, all fecal samples were stored in a -80°C refrigerator after defecation within 6 hours until DNA extraction.

The administration route of antibiotics may also affect the microbial imbalance. The oral antibiotic therapies administered can cause gut dysbiosis and barrier disruption inevitably (Duan et al., 2022). Some studies have shown that parenteral antibiotic delivery doesn’t affect the gut microbiota. However, these findings challenge the theory that parenteral antibiotic delivery does not reach the gut, thereby sparing the gut microbiome (Kelly et al., 2021). Antibiotics, which included *β-*lactam, quinolone, glycopeptide, and lincosamide drugs, were prescribed for patients in the present study. The present study explored the effect of intravenous antibiotics on gut microbiota. The results showed intravenous antibiotics could perturb the composition of the gut microbiota in cardiac surgery patients. The community richness and diversity were significantly decreased when antibiotics were administered over 7 days. This suggests that intravenous antibiotic treatments drastically changed the microbial community. The bacterial community in the intestinal tract is in an ideal equilibrium state, so the disappearance of one “keystone” species may have a great impact on other species. Hence, the effects of antibiotics on the gut microbiota may extend beyond the antibiotic-sensitive bacterial species (Duan et al., 2022). Generally, antibiotics were administrated for a specified and prescribed period, then they were withdrawn. The relative abundance of some bacterial genera cannot recover to the state of pre-antibiotics when antibiotics are withdrawn over 7 days from the aspect of the top 20 bacterial species. The effect of antibiotics on gut microbiota might need antibiotic treatment over 7 days, and the disappearance of the effect of antibiotics on gut microbiota might need antibiotics withdrawn over 14 days. This was consistent with the previous report (Palleja et al., 2018). It had been reported that the effective antibiotic treatment of the gut microbiota showed up after antibiotics treatment over 4 days, and the gut microbiota would recover from a single antibiotic exposure within about 2 weeks in adults. Such as, Palleja et al. (Palleja et al., 2018) reported that after a four-day broad-spectrum antibiotic intervention for healthy volunteers, the species richness and diversity of gut microbiota were dramatically reduced compared to the pre-antibiotic state on the 8^th^ day and 4^th^ day, respectively, and the diversity gradually recovered after antibiotic intervention quit. The recovery ability of the gut microbiota was individualized and related to individual ages, the amount, and frequency of antibiotic treatment (Duan et al., 2022). The days of antibiotic treatment were 17.9 ± 10.3 days in the present study. After all, this phenomenon was due to antibiotics moving the microbiota to alternative stable states and body feedback action (Lozupone et al., 2012).

Antibiotic regimens are varied, and change in antibiotic regimens often occurs in clinical practice. Besides the analysis of total antibiotics, the effects of a single antibiotic on gut microbiota were further explored. All results demonstrated that sulperazone had a significant effect on the composition of gut microbiota. Most cephalosporins are eliminated unchanged in the urine. Nevertheless, approximately 70-80% of cefoperazone (one component of sulperazone) is removed *via* biliary excretion; the remainder is removed *via* renal excretion (Bijie et al., 2005). 4 patients were prescribed levofloxacin, and 2 patients were prescribed moxifloxacin during the analysis of the effect of quinolones on gut microbiota. It was reported that approximately 87% of levofloxacin and 20% of moxifloxacin are excreted through the kidney (Bolon, 2011; Kocsis et al., 2016), and approximately 70% of unchanged tienam and meropenem are in the renal excretions (Salmon-Rousseau et al., 2020). These results demonstrated that merely those antibiotics excreted through bile could reach the intestinal tract and then affect the composition of gut microbiota, however, the antibiotics excreted through the kidney have little effect on gut microbiota for intravenous antibiotics.

Age- and sex-related changes in gut microbiota composition had been explored in the present study. It was reported that *α-*diversity which included *Chao*, *Sobs*, and *Shannon* index was stable during adulthood and then increased in the elderly (Odamaki et al., 2016). This indicates *α-*diversity is higher (Badal et al., 2020) for the elderly and the gut microbiota becomes more diverse and variable with advancing age (Kim and Jazwinski, 2018). Sex-related changes in gut microbiota composition were a contradiction. Some studies reported that the gut microbiota composition was different between females and males (Min et al., 2019; Holingue et al., 2020; Koliada et al., 2021). Haro et al. (Haro et al., 2016) identified the gut microbiota signatures associated with obesity as a function of changes in gender and body mass index (BMI), their results showed that there were no significant differences in *α-* and *β-*diversity between females and males (Haro et al., 2016). The results of age- and sex-related changes in gut microbiota composition were consistent with reported studies in the present study. The effect of antibiotic treatment on gut microbiota in elderly patients was consistent in all patients and the proportion of the elderly population was 57.1% (88 elderly patients) in the present study.

The physiological function of gut microbiota was attempted to merely explore the sulperazone treatment in the present study because other single antibiotics did not affect both *α-* and *β-*diversity. We focused on menaquinone biosynthesis by gut microbiota, because most patients underwent heart valve replacement surgery, and these patients need to be administered warfarin for the prevention and/or treatment of thromboembolism after surgery. Warfarin acts as anticoagulant by interfering with the recycling of vitamin K. Vitamin K is a group of fat-soluble vitamins, and includes two major forms: phylloquinone (vitamin K1, VK1) and menaquinone (vitamin K2, VK2). VK1 is found exclusively in plants, while VK2 (except MK-4) is mainly produced by a series of congeners synthesized by gram-positive bacteria in the human gastrointestinal tract (Merli and Fink, 2008). The human gastrointestinal tract as a VK2 reservoir is estimated to contribute 10–50% of the human vitamin K requirement. The main sources of VK2 from gut microbiota include *Bacteroides*, *Prevotella*, *Bifidobacterium*, *Eubacterium lentum*, *Enterobacter*, *Veillonella*, *Lactococcus lactis*, *Leuconostoc*, *Streptococcus, Staphylococcus aureus*, *Escherichia*, *Klebsiella*, and *Propionibacteria* (Karl et al., 2015; Biesalski, 2016; Linares et al., 2017). Changes in the composition of gut microbiota may also affect the anticoagulant effect of warfarin. The pathway modules of menaquinone include menaquinone biosynthesis (M00116), futalosine pathway (M00930), and modified futalosine pathway (M00931). Some enzymes involved in menaquinone biosynthesis were decreased post-sulperazone treatment. The relative abundances of *Bacteroides* and *Prevotella* have significantly reduced post-sulperazone treatment (**Figure S8C**). The result demonstrates that sulperazone might affect coagulation function. Combined sulperazone had an inverse correlation with warfarin stable dosage in the previous report (Li et al., 2019).

There were also some limitations of the present study. The composition of the gut microbiota is different in different gastrointestinal tract sites. Fecal samples were detected to learn about the gut microbiota in clinical practice, which merely reflected the composition of gut microbiota in the sigmoid colon lumen for patients (Tiihonen et al., 2010). Patients have often prescribed probiotics after surgery. Probiotics have been demonstrated to have beneficial effects on the health of the host person (Angelucci et al., 2019). The effect of probiotics on gut microbiota was not considered in the present study.

In conclusion, the intravenously administered antibiotics which are excreted *via* bile could disturb the composition of the gut microbiota. The effect of antibiotics on gut microbiota is produced by antibiotics treatments over one week. The recovery of gut microbiota to the state of pre-antibiotics may require over two weeks of antibiotics withdrawal. The richness and diversity of gut microbiota are more in the elderly when compared with other age groups. There is no difference in gut microbiota composition between the sexes. Long-term use of sulperazone may affect coagulation function.

## Data availability statement

The partial data presented in the study are deposited in the Sequence Read Archive repository, BioProject ID: PRJNA923595. Further inquiries can be directed to the corresponding author/s.

## Ethics statement

The studies involving human participants were reviewed and approved by the health authority ethics committee of the First Affiliated Hospital of Soochow University. The patients/participants provided their written informed consent to participate in this study.

## Author contributions

LX, ZS, BS, and LM designed the study. LX, YD, and QQ performed the study. LX analyzed the data and drafted the manuscript. YD, QQ, LL, XD, and YaZ interpreted the data. YiZ, KL, RS, KS, AD, BS, and LM reviewed the manuscript. All authors contributed to the article and approved the submitted version.
