# Supplementary material for: Assessment of the impact of intravenous antibiotics treatment on gut microbiota in patients: Clinical data from pre-and post-cardiac surgery
Source: Front Cell Infect Microbiol. 2023 Jan 19;12:1043971. doi: 10.3389/fcimb.2022.1043971 (PMC9896080; doi:10.3389/fcimb.2022.1043971)
Supplement: Supplementary file 1 [file Image_1.pdf]

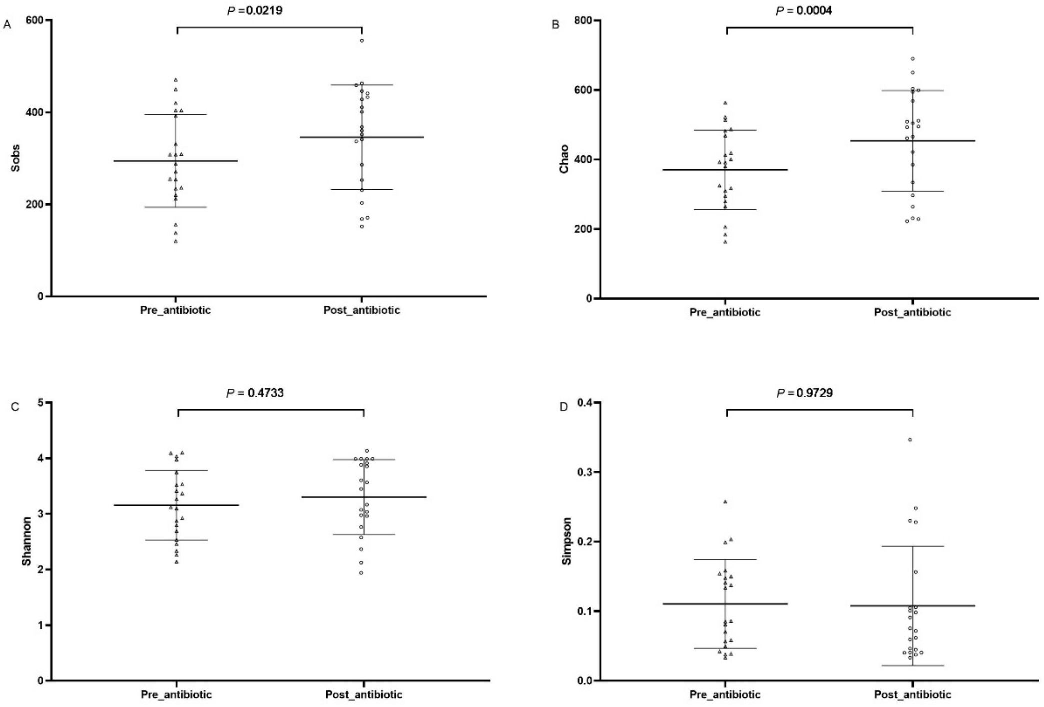

Figure S1. The community richness and diversity for cefuroxime treatment. A: *Sobs* index; B: *Chao* index; C: *Shannon* index; D: *Simpson* index.

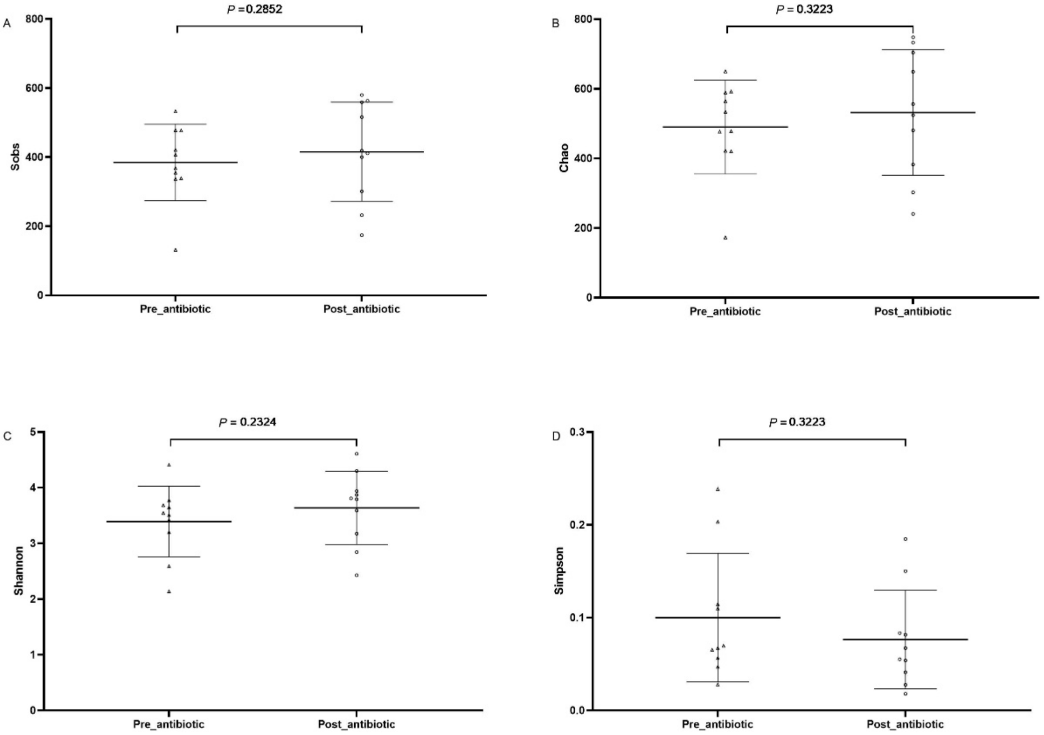

Figure S2. The community richness and diversity for ceftazidime treatment. A: *Sobs* index; B: *Chao* index; C: *Shannon* index; D: *Simpson* index.

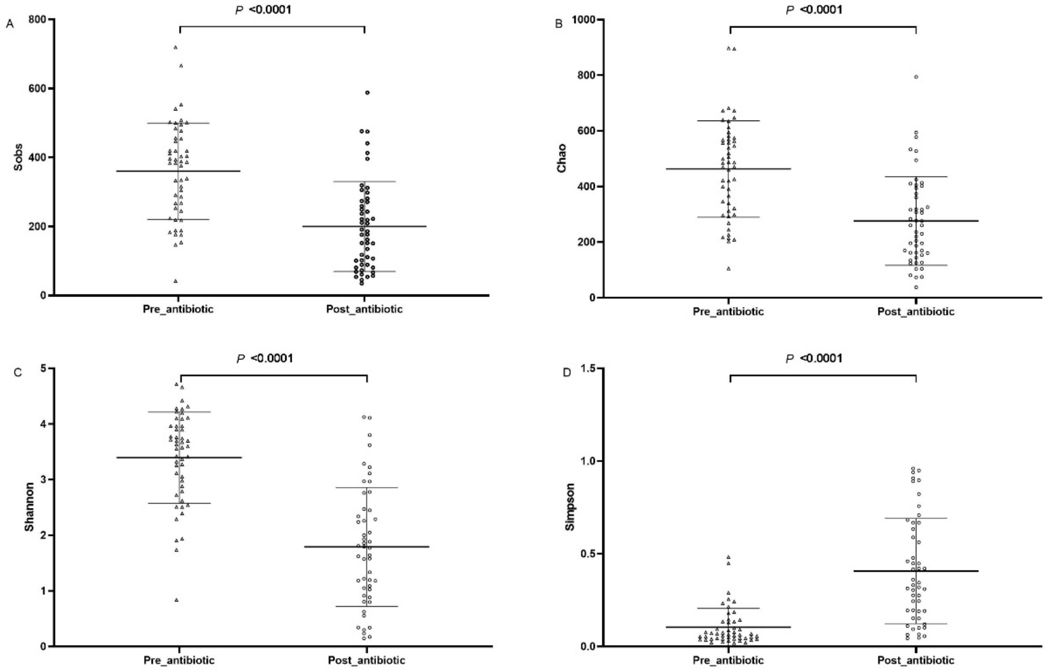

Figure S3 The community richness and diversity for sulperazone treatment. A: *Sobs* index; B: *Chao* index; C: *Shannon* index; D: *Simpson* index.

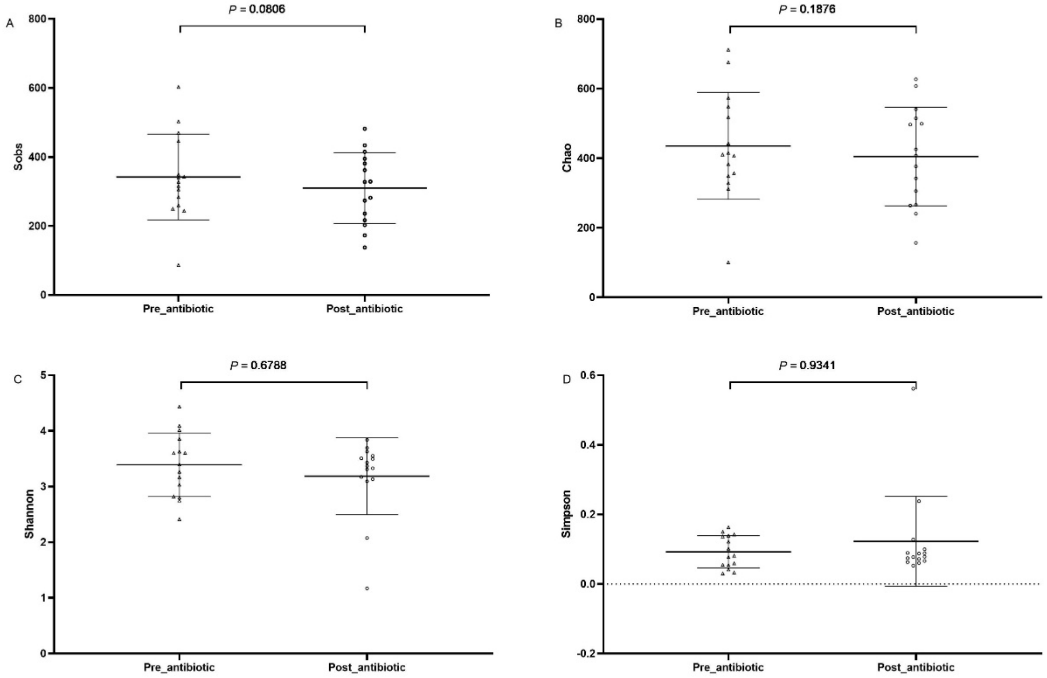

Figure S4. The community richness and diversity for tienam treatment. A: *Sobs* index; B: *Chao* index; C: *Shannon* index; D: *Simpson* index.

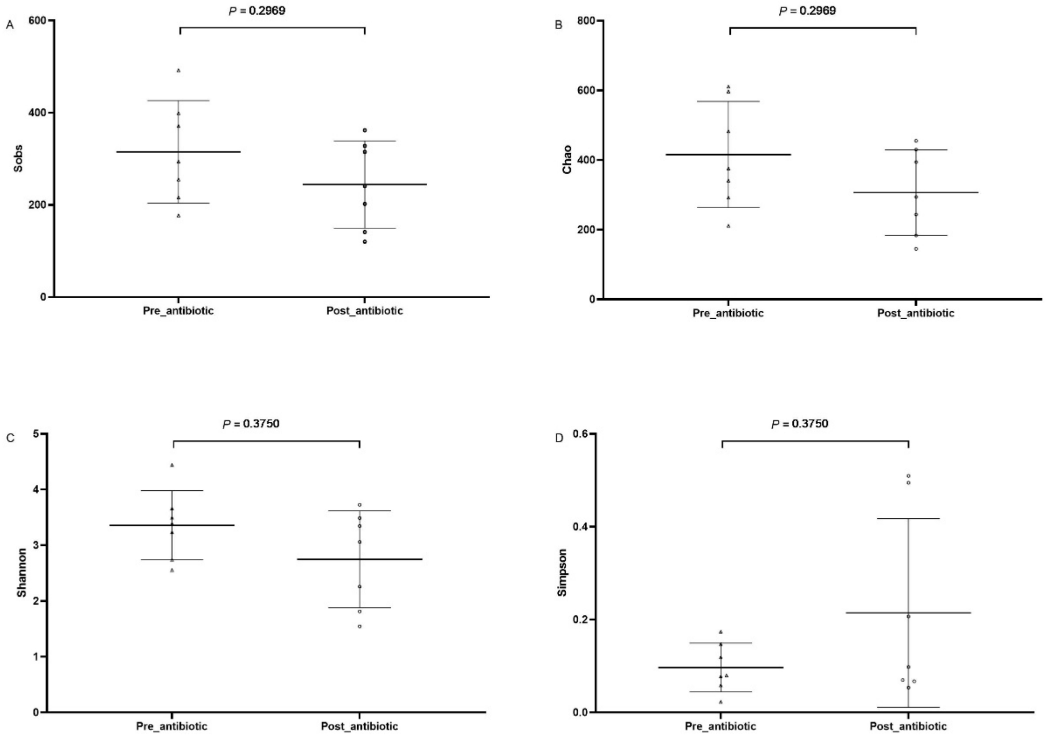

Figure S5. The community richness and diversity for meropenem treatment. A: *Sobs* index; B: *Chao* index; C: *Shannon* index; D: *Simpson* index.

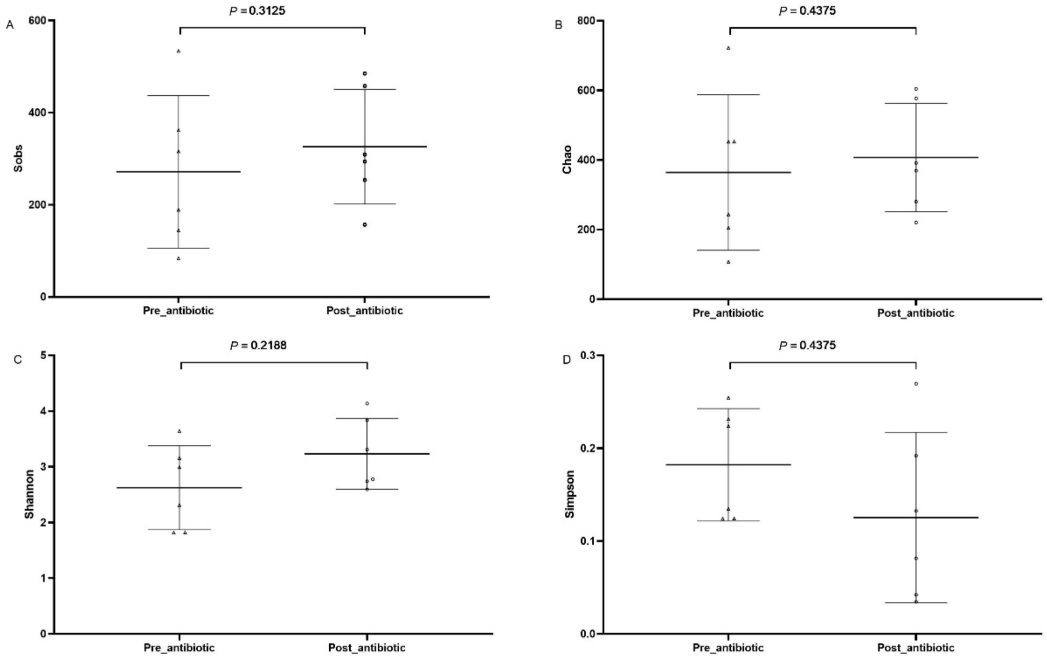

Figure S6. The community richness and diversity for quinolones treatment. A: *Sobs* index; B: *Chao* index; C: *Shannon* index; D: *Simpson* index.

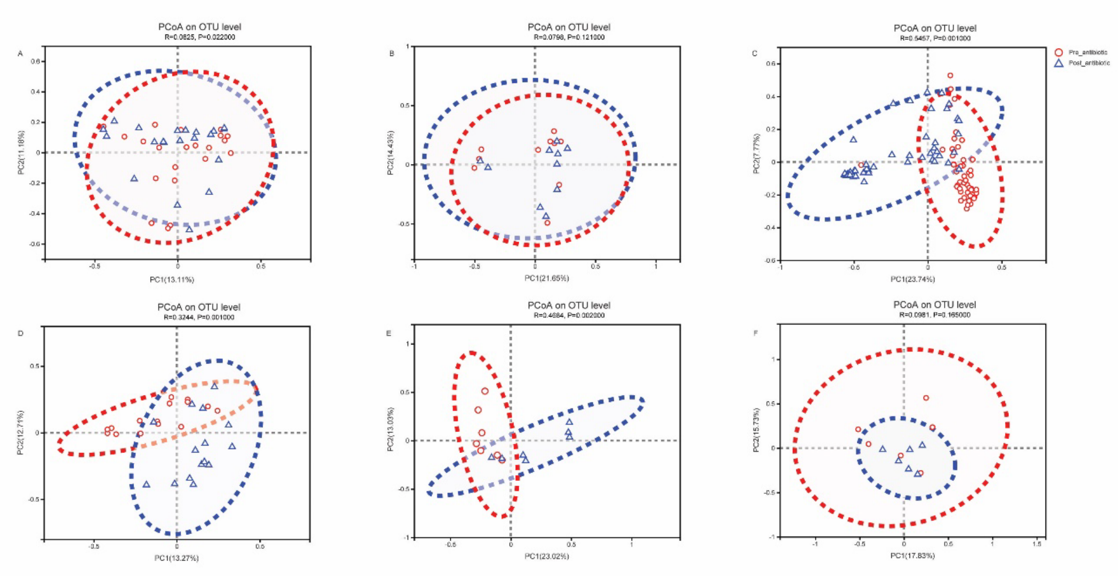

Figure S7. PCoA (principal coordinates analysis) of gut microbiota for different single antibiotic treatments. A: cefuroxime; B: ceftazidime; C: sulperazone; D: tienam; E: meropenem; F: quinolones. The dotted line represents the 95% confidence interval line.

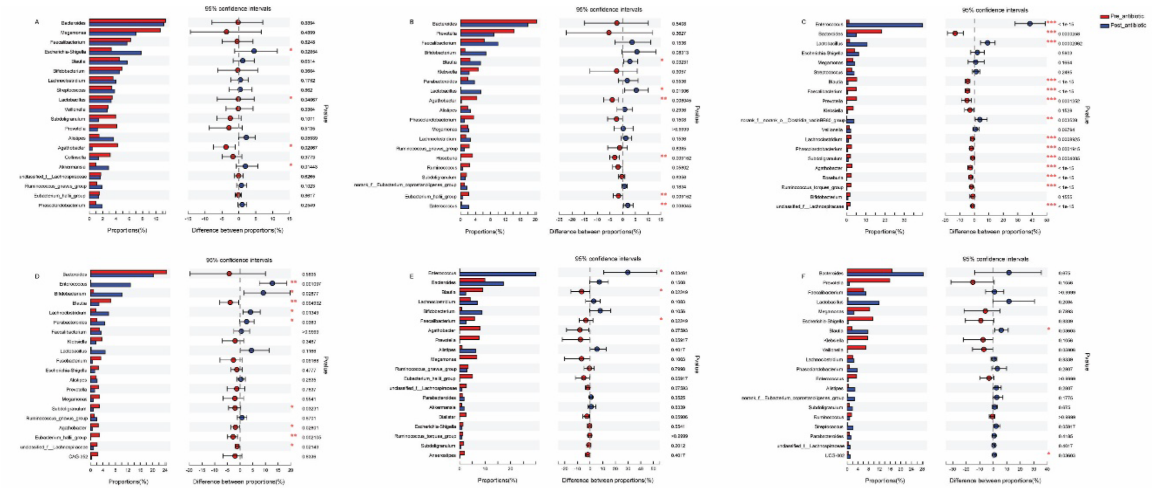

Figure S8. Comparison of the top 20 bacterial species for different single antibiotic treatments. A: cefuroxime; B: ceftazidime; C: sulperazone; D: tienam; E: meropenem; F: quinolones.

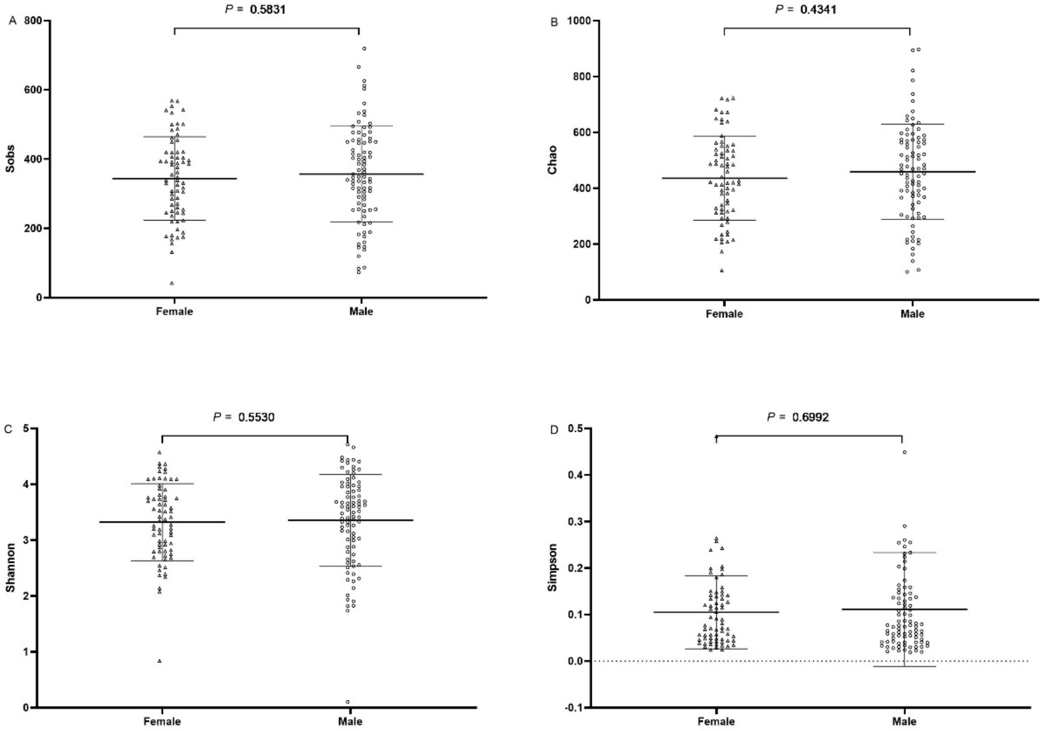

Figure S9. The community richness and diversity of gut microbiota for sex. A: *Sobs* index; B: *Chao* index; C: *Shannon* index; D: *Simpson* index.

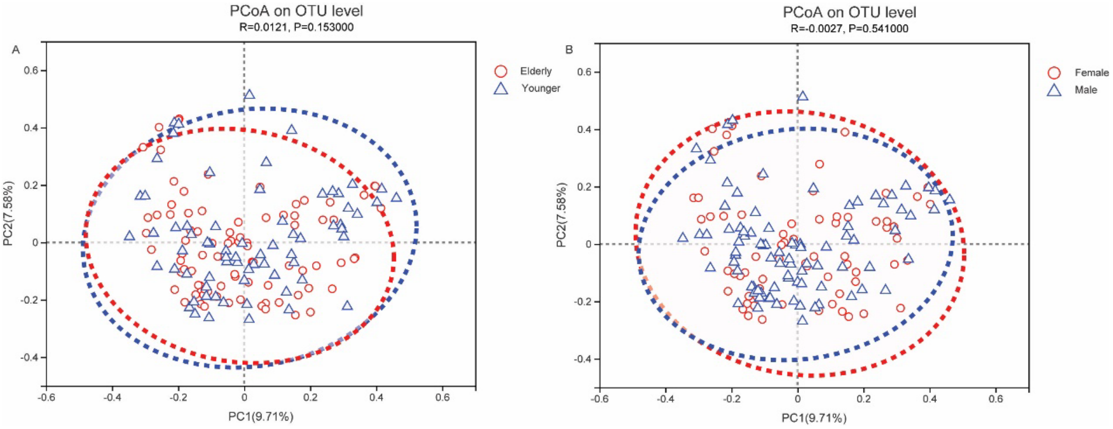

Figure S10. PCoA (principal coordinates analysis) of gut microbiota for age and sex. A: age; B: sex. The dotted line represents the 95% confidence interval line.

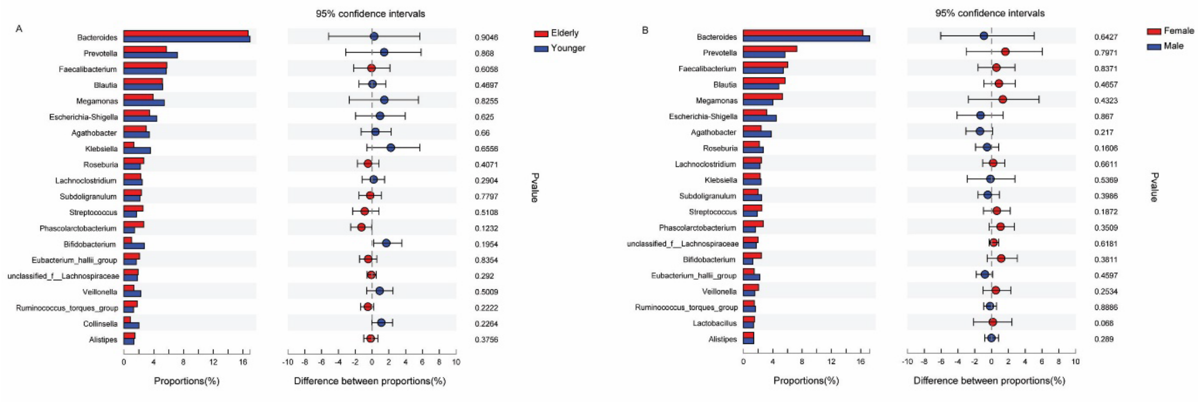

Figure S11. Comparison of the top 20 bacterial species between age and sex. A: age; B: sex.

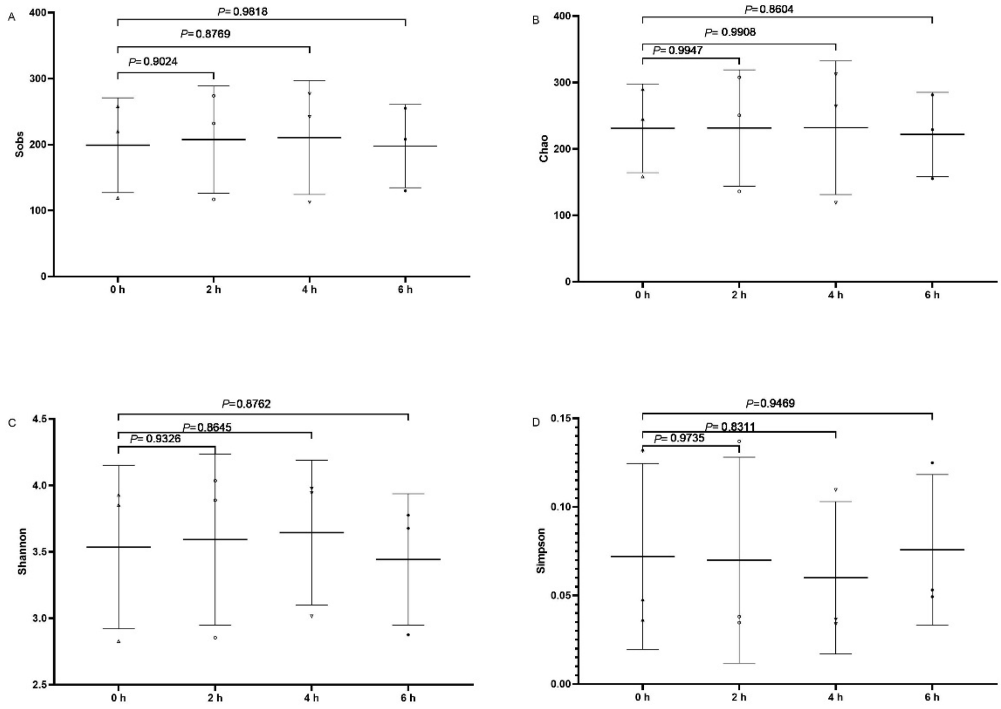

Figure S12. The community richness and diversity of gut microbiota for healthy volunteers. A: *Sobs* index; B: *Chao* index; C: *Shannon* index; D: *Simpson* index.

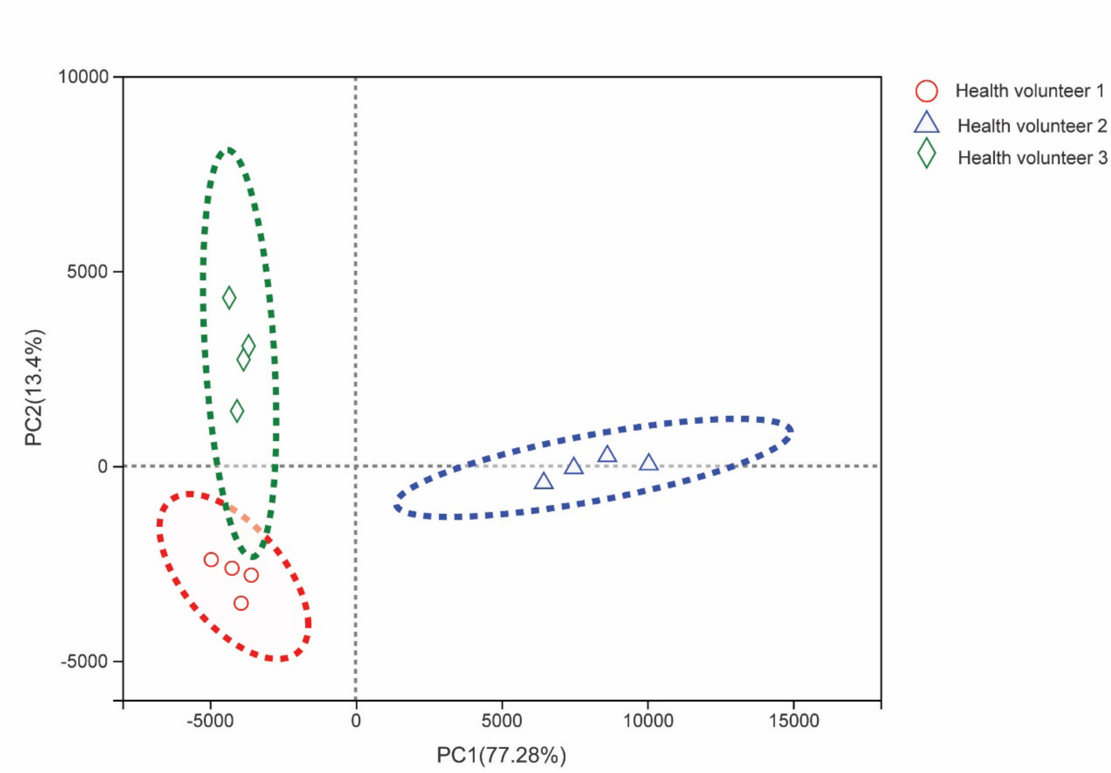

Figure S13. PCoA (principal coordinates analysis) of gut microbiota for healthy volunteers. The dotted line represents the 95% confidence interval line.

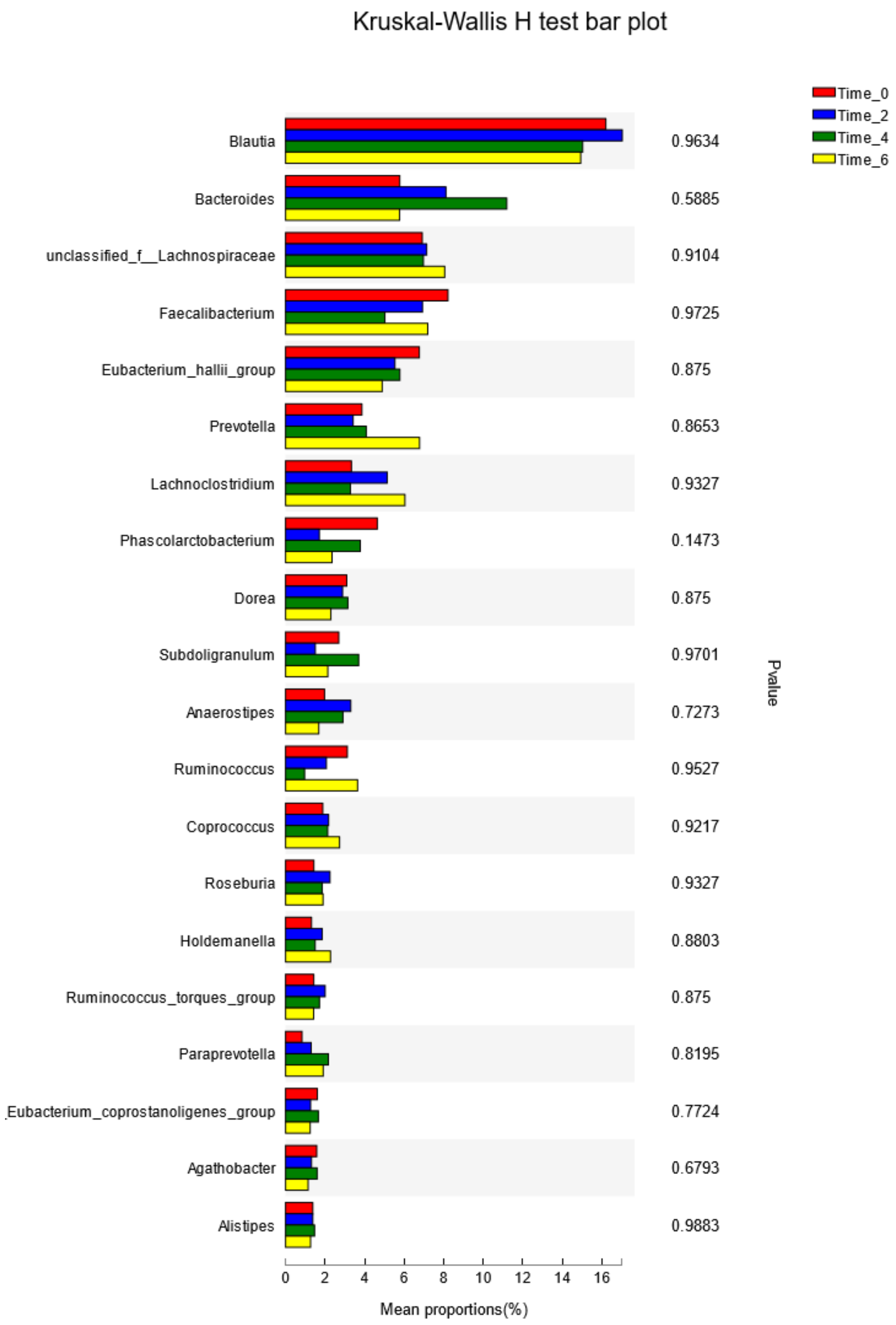

Figure S14. Comparison of the top 20 bacterial species for healthy volunteers.

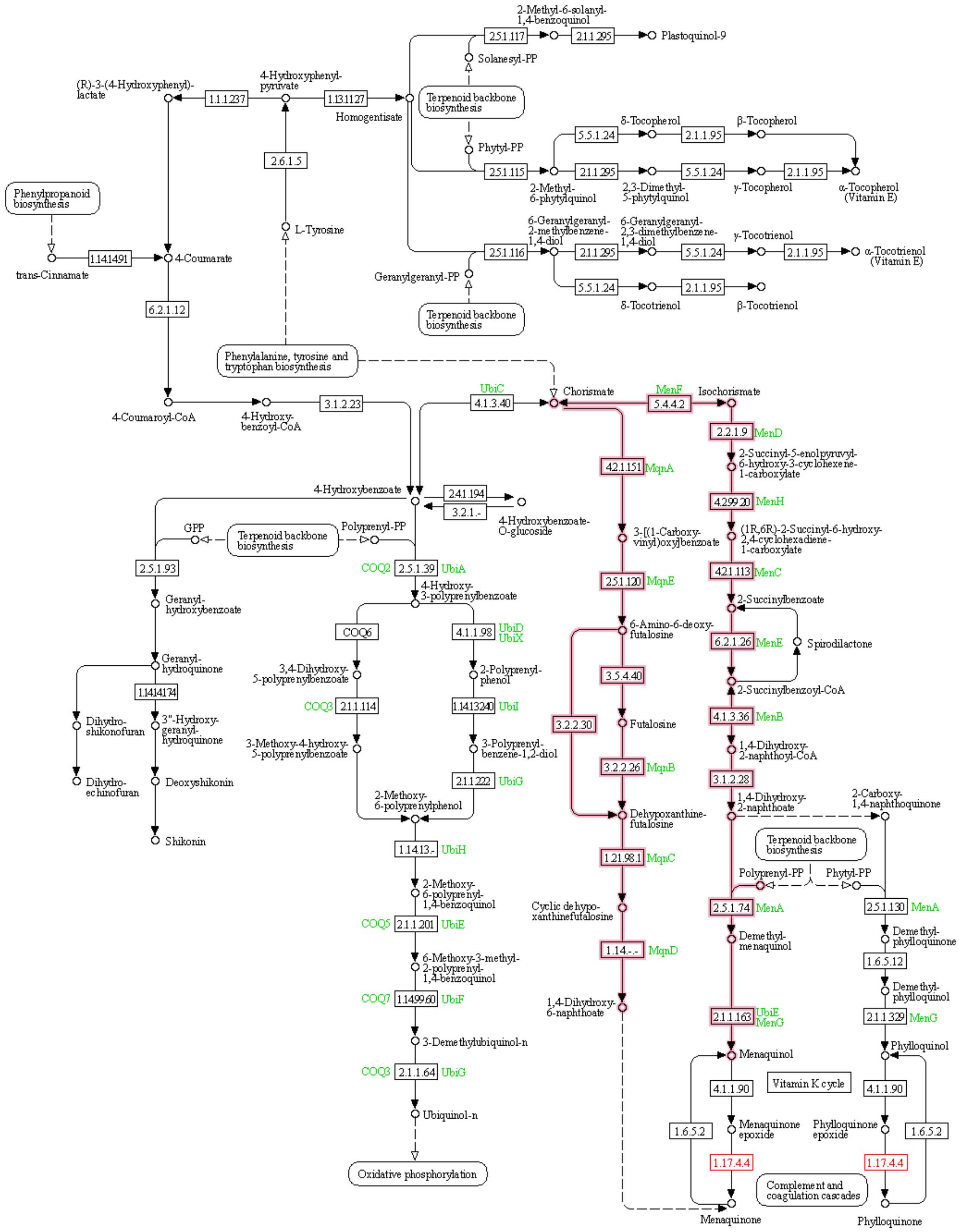

Figure S15. The pathway modules of menaquinone biosynthesis with the highlighted red line.
